# Horizontal histological sections in the preliminary evaluation of basal cell carcinoma submitted to Mohs micrographic surgery^☆^^☆☆^

**DOI:** 10.1016/j.abd.2017.11.001

**Published:** 2019-10-26

**Authors:** Poliana Santin Portela, Danilo Augusto Teixeira, Carlos D́Aparecida Santos Machado, Maria Aparecida Silva Pinhal, Francisco Macedo Paschoal

**Affiliations:** a Graduate Program in Health Sciences, Faculdade de Medicina do ABC, Santo André, SP, Brazil; b Department of Dermatology, Hospital de Doenças Tropicais, Goiânia, GO, Brazil; c Discipline of Dermatology, Faculdade de Medicina do ABC, Santo André, SP, Brazil; d Discipline of Biochemistry, Faculdade de Medicina do ABC, Santo André, SP, Brazil

**Keywords:** Carcinoma, basal cell, Mohs surgery, Pathology

## Abstract

**Background:**

Mohs micrographic surgery is a surgical technique for the treatment of nonmelanoma skin cancer. Surgery begins by removing the visible tumor before excision of the tissue specimens for evaluation of the tumor margins.

**Objectives:**

To present a new way to evaluate the material obtained from debulking, by horizontal histological analysis of the fragment.

**Methods:**

Descriptive retrospective cross-sectional study based on the medical records and histological lamellae of patients with primary basal cell carcinomas smaller than 1.5 cm submitted to Mohs micrographic surgery and who had the visible tumor analyzed by horizontal histological sections.

**Results:**

The sample evaluated included 16 patients with lesions located on the face. Comparing the histopathological examinations of incisional biopsy in vertical sections and debulking in horizontal sections, there was agreement in seven cases. The histological analysis performed in horizontal sections allowed identification of the tumor site in 13 cases, and the relation between tumor and margin showed that in 11 cases, the lateral margin was compromised.

*Study limitations*: The technique was better-applied in lesions smaller than 2 cm.

**Conclusion:**

Horizontal histological analysis of debulking has advantages for Mohs surgery, since it allows visualization of almost all tumor extension in the same view plane of the dermatoscopy, allowing better definition of the histological subtype, tumor site, and tumor/margin of lesions less than 1.5 cm.

## Introduction

Mohs micrographic murgery (MMS) is a surgical technique for the treatment of non-melanoma skin cancer.^1^ It consists of a series of standardized steps with precise and complete histological control of tumor margins, with superior cure rates, and maximum preservation of normal tissue in relation to conventional surgery.^2^ The correlation between the presence of tumor in the histological examination and its correct location on the surgical map is essential for complete resection of the lesion and preservation of normal tissue.^1^

The success of micrographic surgery is inherently linked to the reliability of each of the many steps that make up the technique.^3^ Since its first description by Frederic E. Mohs,^4^ micrographic surgery has been undergoing a constant process of modifications and adaptations, with the objective of developing technical variations that best adapt to the daily routine of dermatologic surgeons. However, the basic steps of the procedure are preserved^4^:

(1) Tumor removal;
(2) Delimitation of a margin ranging from2–5 mm depending on histological type and tumor location;
(3) Removal of thin tissue layer containing the lateral margins and tumor bed;
(4) Mapping of the surgical specimen;
(5) Microscopic analysis with total control of margins;
(6) Selective excision of areas with residual tumor;
(7) End of the excision after obtaining free margins and posterior reconstruction of the surgical wound.

Before surgical procedure begins, the tumor site is identified and marked with a dermatoscope. This allows the precise delimitation of the visible tumor and the creation of a surgical margin of 1–5 mm, depending on tumor type. Surgery begins with debulking or enucleation, involving removal of the visible tumor prior to excision of tissue specimens for evaluation of tumor margins. There are different ways of performing enucleation. Some surgeons make visible tumor curettage in order to better delimitate the margins, others opt to histologically evaluate the excised material.^5^

Traditionally, the histological analysis of the material from enucleation is performed by conventional incisions, *i.e.*, in vertical transverse sections. This is made for documentation and to identify specific tumor growth pattern within the tissue, especially if the biopsy was not definitive.^5^ However, the disadvantages of this analysis fall under the same conditions of the conventional pathology, where only a very small sample of the tumor is studied.^6^ Serial vertical incisions are made at 2–4 mm intervals in the conventional method of evaluation (bread-loaf method). This leaves marginal areas between sections that are not microscopically visualized, and less than 1% of the tumor margins are evaluated.^5^

Based on reflectance confocal microscopy, where images are parallel to the surface at horizontal orientation, which allows a broader analysis of the tumor architecture, this study aimed to present a new way of evaluating the tumor enucleation material from the horizontal histological analysis of the fragment resulting from the removal of the visible tumor obtained by enucleation in the first step of MMS.^7^, ^8^

## Objectives

To compare the histological type of horizontal histological analysis of the enucleation with the vertical histological type of the preoperative biopsy and to evaluate the relation of the tumor with the margin of tumor resection.

## Methods

This was a descriptive retrospective cross-sectional study based on the medical records and histological lamellae of patients with primary basal cell carcinomas (BCCs) smaller than 1.5 cm submitted to MMS and who had the visible tumor analyzed by horizontal histological sections. The study was submitted and approved by the ethics committee. Epidemiological data were collected from patients’ records, such as sex, age, histological type defined by previous biopsy by the pathologist, histological type defined by horizontal histological incision, tumor size, initial and final size of the surgical defect, number of phases necessary for complete surgical excision, and type of reconstruction adopted. Data were collected and entered into an Excel table. The histological slides from surgeries were reviewed and analyzed, defining the histological subtype of the tumor and its proximity to the excised margins.

In all cases, the following steps were performed:

(1) Preoperative delineation (or marking) of the tumor margins with a lighting and image magnification instrument, the dermatoscope 3GEN DermLite II hybrid m.
(2) After the steps of asepsis, antisepsis, and infiltrative anesthesia, the surgical procedure began with the enucleation stage. This step consists of the excision of a thin layer of the entire visible lesion (area delimited by dermoscopy) for horizontal histological analysis.
(3) After enucleation, a margin of 2 mm was added to the area that was removed and other MMS stages were performed.
(4) Horizontal histological sections were taken from the surface to the depth and the authors evaluated data such as histological subtype, initial tumor size, and tumor-margin relationship.

**Figure 1:**
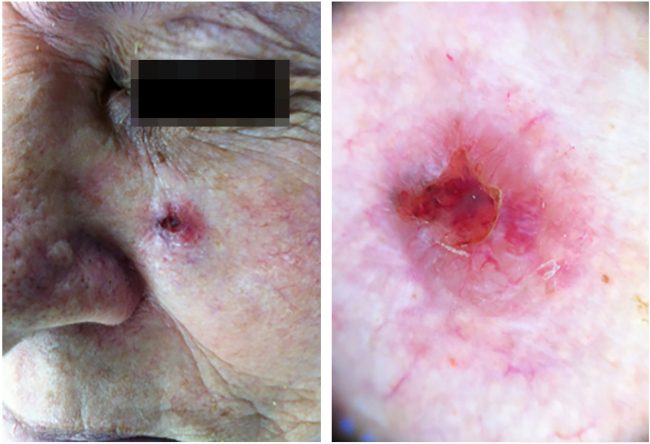
Clinical and dermatoscopic aspect (×20 magnification) of basal cell carcinoma located in the left malar region.

**Figure 2:**
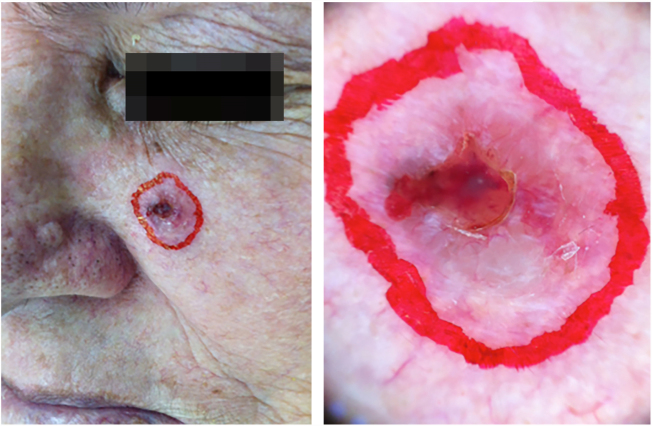
Tumor delimitation with dermatoscope.

**Figure 3:**
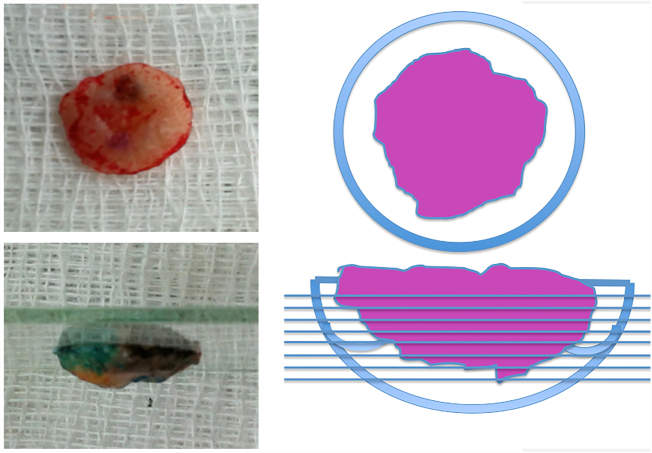
Debulking image and demonstration of how the incisions were made (horizontal incisions from the surface to the depth of the tumor).

**Figure 4:**
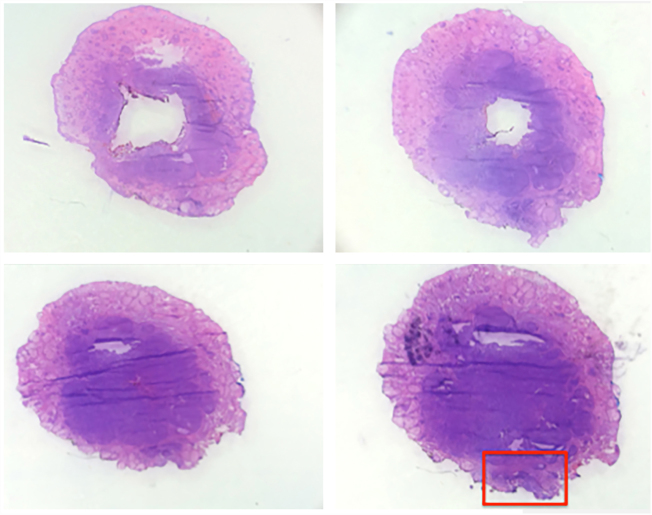
Horizontal histological sections of the debulking (hematoxylin-eosin, ×20). Top left image corresponding to the surface incision and lower right image to the deepest incision. Highlighted lateral margin involvement (red rectangle).

**Figure 5:**
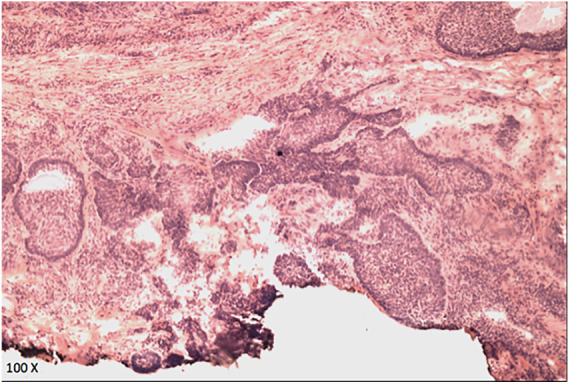
Microscopic view of the horizontal histological incision with compromised margin (hematoxylin-eosin, ×100).

The case presented in Figure 1, Figure 2, Figure 3, Figure 4, Figure 5 illustrates the step-by-step in each of the evaluated cases.

## Results

The sample evaluated included 16 patients, four males and 12 females, aged between 50 and 84 years (Table 1). All lesions were located on the face, all of them smaller than 1.5 cm in diameter. The predominant subtype of BCC in preoperative biopsy analysis was nodular or solid form (six cases), followed by infiltrative sclerodermiform (four cases), nodular and micronodular (four cases), and micronodular (two cases). Comparing the histopathological examinations of incisional biopsy in vertical sections and the debulked piece in horizontal sections, there was agreement in seven cases and disagreement in nine cases. Vertical histopathological analysis (preoperative punch biopsy) was incorrect in four cases of the 16 evaluated and failed to identify infiltrative forms of BCCs in two cases, which were diagnosed as nodular and micronodular BCCs, but in the horizontal histological analysis of the debulking, they corresponded to micronodular BCCs. In these cases, even though there was no total agreement, the preoperative biopsy diagnosed the micronodular infiltrative portion. In one of the slides evaluated by horizontal incisions, it was not possible to identify the presence of tumor tissue.

**Table 1:** Clinical data, histological findings, and tumor characteristics of the 16 cases analyzed

Case	Sex	Age (years)	Location	Size (cm)	BCC subtype of previous biopsy (histology)	BCC subtype of debulking (histology)	Number of MMS phases	Involvement of lateral margins	Adequate identification of tumor site
1	M	64	Forehead	0.8 × 0.7	Sclerodermiform	Sclerodermiform	2	Involved	Yes
2	F	82	Cheek	1.9 × 1.9	Nodular	Nodular	1	Involved	Yes
3	F	57	Inferior eyelid	2.0 × 1.5	Nodular	Micronodular	1	Involved	Yes
4	M	51	Inferior eyelid	1.0 × 0.8	Nodular	Nodular	1	Involved	Yes
5	F	69	Nose	0.6 × 0.5	Micronodular	Micronodular	1	Free	Yes
6	F	64	Nose	0.8 × 0.7	Nodular and micronodular	Micronodular	1	Involved	Yes
7	F	84	Nose	0.7 × 0.6	Sclerodermiform	Sclerodermiform	1	Involved	Yes
8	M	53	Nose	1.0 × 1.0	Nodular	No tumor	1	Free	No
9	F	64	Nose	1.3 × 0.9	Sclerodermiform	Micronodular	1	Involved	Yes
10	F	79	Nose	0.8 × 0.7	Nodular and micronodular	Micronodular	5	Free	Yes
11	F	50	Inferior eyelid	0.9 × 0.6	Nodular and micronodular	Micronodular	2	Involved	Yes
12	M	70	Nose	1.2 × 1.2	Sclerodermiform	Superficial	2	Free	No
13	F	79	Cheek	0.7 × 0.6	Nodular and micronodular	Nodular	2	Involved	Yes
14	F	68	Nose	0.4 × 0.3	Micronodular	Micronodular	2	Involved	No
15	F	60	Nose	1.0 × 0.7	Nodular	Nodular	2	Involved	Yes
16	F	63	Inferior eyelid	0.45 × 0.45	Nodular	Micronodular	1	Involved	Yes

BCC, basal cell carcinoma; MMS, Mohs micrographic surgery.

The histological analysis of the visible tumor of each patient performed by horizontal incisions allowed identification of the tumor site in 13 cases, and the relation between tumor and margin showed that in 11 cases, the lateral margin was compromised. Eight cases needed only one surgical phase, six patients had to undergo a second phase, and one patient required four phases for total BCC excision.

## Discussion

Traditional pre-operative biopsies of cutaneous malignancies are performed to provide accurate diagnosis of clinically diagnosed tumors and hence to indicate the best treatment. When these tumors are definitively treated by simple excision, the piece is sent to the pathology department and the general architecture of the central tumor nodule is further histologically evaluated, during which any inconsistencies are observed. If the same tumor is excised through Mohs technique, the surgical margin receives complete evaluation by the surgeon himself, allowing the preservation of tissue, resulting in higher cure rates. However, the tumor itself will never be seen if the first phase of the Mohs surgery has tumor-free margins, and the preoperative biopsy is the only sample of the actual tumor. In rare cases, initial biopsy sampling error and/or limitations in the dermatopathologist's ability to assess the overall morphology of a tumor based on a small biopsy may limit the ability to make a correct diagnosis. This may have diagnostic and/or therapeutic implications.^6^

When the visible portion of the BCC is removed by curettage or excision of the tumor for conventional histological evaluation, the question is whether or not some tumor characteristic is missed by not performing the complete histological analysis. In most cases, the answer to this question is no.^6^ However, the horizontal incision serves as an important guideline in the planning process of MMS or in subsequent phases. In this way, the surgeon has a vision of 100% of the tumor, and thus can define more characteristics, such as its histological subtype, lateral margin involvement, and lateral growth pattern.

An important question is this: What steps can be taken to minimize diagnostic inadequacies prior to Mohs surgery?^6^ One way to minimize the diagnostic inaccuracies for the Mohs surgeon is to evaluate the intraoperative debulking specimens obtained in horizontal sections. Horizontal histological analysis, *i.e*., parallel to the surface of the epidermis, provides a better study of tumor shape, being able to map it and better delimit the surgical margin. This is because some subtypes of BCCs have small extensions or roots that may not be seen in conventional vertical incisions.^9^

## Conclusion

MMS has come to minimize diagnostic inaccuracies while providing the patient with optimal treatment for many cutaneous neoplasms. Horizontal histological analysis of debulking has advantages for Mohs surgery, since it provides the same view of the tumor as confocal microscopy and dermoscopy, allowing better definition of the histological subtype, a more definite view of the tumor site and, in lesions smaller than 1.5 cm, visualization of the entire tumor, providing a good idea of the tumor's relationship with the margin of the lesion.

## Financial support

None declared.

## Author's contribution

Poliana Santin Portela: Conception and planning of the study; elaboration and writing of the manuscript; obtaining, analyzing and interpreting the data; intellectual participation in propaedeutic and/or therapeutic conduct of the cases studied; critical review of the literature; critical review of the manuscript.

Danilo Augusto Teixeira: Elaboration and writing of the manuscript; intellectual participation in propaedeutic and/or therapeutic conduct of the cases studied.

Carlos D́Aparecida Santos Machado: Effective participation in research orientation.

Maria Aparecida Silva Pinhal: Effective participation in research orientation.

Francisco Macedo Paschoal: Approval of the final version of the manuscript; conception and planning of the study; obtaining, analyzing and interpreting the data; effective participation in research orientation; intellectual participation in propaedeutic and/or therapeutic conduct of the cases studied; critical review of the literature; critical review of the manuscript.

## Conflicts of interest

None declared.
